# Initial experience with prostatic urethral lift versus enucleation of the prostate: a retrospective comparative study

**DOI:** 10.1186/s12894-023-01366-8

**Published:** 2023-11-18

**Authors:** Daisuke Obinata, Rio Uehara, Sho Hashimoto, Ken Nakahara, Tsuyoshi Yoshizawa, Junichi Mochida, Kenya Yamaguchi, Satoru Takahashi

**Affiliations:** Department of Urology, Nihon University School of Medicine, 30-1, Oyaguchikamicho, Itabashi-ku, Tokyo, 173-8610 Japan

**Keywords:** Urolift, Prostatic urethral lift, Bipolar transurethral enucleation of the prostate, Benign Prostatic Hyperplasia, International prostate symptom score, Core lower urinary tract symptom score

## Abstract

**Background:**

This study aimed to assess initial results and patient characteristics of prostatic urethral lift (PUL) compared with those of bipolar transurethral enucleation of the prostate (TUEB) in the treatment of benign prostatic hyperplasia (BPH) in older patients.

**Methods:**

This retrospective study was conducted at a single institution and involved 25 consecutive patients with BPH who underwent PUL between April 2022 and May 2023. Patient characteristics, operative details, and pre- and postoperative symptom scores were evaluated. The results were compared with those of a previously reported TUEB group (n = 55).

**Results:**

The mean age of the patients in the PUL group was 74.6 years, and the mean prostate volume was 47.5 ml. The PUL procedure significantly improved urinary symptoms, particularly incomplete emptying (*p* = 0.041), intermittency (*p* = 0.005), and weak stream (*p* = 0.001). The PUL group had higher comorbidity scores (*p* = 0.048) and included older patients (*p* = 0.002) than the TUEB group. TUEB showed better improvements in some symptoms and maximum flow rate (*p* = 0.01) than PUL; however, PUL had a shorter operative time and fewer complications than TUEB (*p* < 0.001).

**Conclusion:**

The initial results demonstrate the efficacy and safety of PUL in older patients with BPH. Despite TUEB showing better outcomes in certain aspects than PUL, PUL offers advantages such as shorter operative time and fewer complications. Therefore, PUL can be considered a viable option for high-risk older patients with BPH.

**Supplementary Information:**

The online version contains supplementary material available at 10.1186/s12894-023-01366-8.

## Background

Benign prostatic hyperplasia (BPH) frequently occurs in middle-aged and older men and results in lower urinary tract symptoms (LUTSs) [1]. Generally, most cases of BPH/LUTS can be treated with medications; however, > 25% of patients cease treatment owing to drug side effects or inadequate remission, although a slight improvement is observed in urinary symptoms 1 year after initiating treatment [2, 3]. Surgery for BPH is recommended to prevent bladder dysfunction and renal impairment when drug therapy is ineffective; moderate to severe symptoms are present; and complications such as urinary retention, urinary tract infection, hematuria, and bladder stones are present. Surgical treatment can be divided into three major categories: (a) resection (ablation, ablation) or vaporization of tissues, (b) thermal coagulation or denaturation of tissues, and (c) other techniques such as high-density focused ultrasound therapy. According to the Ministry of Health, Labour and Welfare’s National Database Open Data in Japan, approximately 30,957 BPH surgeries were reported in 2015; however, this number decreased to approximately 26,763 in 2019 [4]. As of October 2021, the older population in Japan has reached 40%, the highest proportion since 1950 (https://www.stat.go.jp/data/jinsui/2021np/index.html). These data indicate that older individuals with surgical indications are more likely to avoid invasive prostate surgery.

Prostatic urethral lift (PUL) is less invasive compared with conventional prostate surgery [5]. In Japan, insurance coverage for PUL has become available since April 1, 2022. However, there are no reports on the initial results of PUL in Japan. Furthermore, in Japan, indications for this procedure are limited to high-risk cases of conventional prostate surgery.

Insurance approval was granted for bipolar transurethral enucleation of the prostate (TUEB) in Japan in 2018; therefore, PUL and TUEB are the most recently approved surgical options in the country. TUEB is associated with fewer bleeding complications than standard transurethral resection of the prostate (TURP), even when the prostate volume is large [6]. However, we previously reported the initial treatment outcomes of TUEB, which is characterized by a longer operative time and higher incidence of urethral stricture and infection compared with TURP [7]. Thus, in this study, we assessed the initial results and patient characteristics of PUL using the UroLift®2 system (Teleflex Medical Japan, Tokyo, Japan) in comparison with those of previous TUEB cases [7] conducted at the same institution.

## Materials and methods

The selection and exclusion criteria were based on the guidelines issued by the Japanese Urological Association, the Japanese Continence Society, and Japanese Society of Endourology and Robotics. Briefly, the indications for PUL, in addition to the general surgical indications for BPH, include high-risk cases that are not well-suited for traditional surgical methods because of overall health conditions. Patients with a prostate volume exceeding 100 ml and those with significant hematuria are considered ineligible for surgery. However, in September 2023, transurethral water vapor therapy became an available option for these indications in Japan; therefore, this procedure was introduced at our facility in November 2022. At our institution, we performed PUL in 26 consecutive patients with BPH between April 2022 and May 2023. After November 2022, we presented the option of water vapor therapy and chose PUL through shared decision-making for a total of 16 cases. One case was excluded from the evaluation because the patient did not return for the follow-up examination. PUL was performed using the UroLift®2 system, a 2.9-mm 0° cystoscope and a perfusion system. The cystoscope was attached to the delivery handle using an implant cartridge. A ventral position within the prostatic urethra at least 1.5 cm distal to the bladder neck was selected, the cystoscope handle was tilted approximately 10° to the patient’s midline, and the implant was inserted into the prostate. Multiple implants were used to ensure opening of the anterior urethra. We evaluated age, Charlson Comorbidity Index score, Geriatric 8 (G8) score, operative time, perioperative complications, and number of implants inserted. In addition, the International Prostate Symptom Score (IPSS), Overactive Bladder Symptom Score (OABSS), Core Lower Urinary Tract Symptom Score (CLSS), and urinary function assessed by single voiding volume, maximum flow rate (MFR), and post-void residual (PVR) were evaluated before (pre) and 1 month after PUL (post). The median follow-up duration was presented in two ways: from pre-to post-surgery (median 2.6; interquartile range (IQR) 2.1–3.0 months), and from surgery to post-surgery (median, 1.2; IQR 1.0–1.3 months). Moreover, we compared our results with previously reported TUEB outcome data (n = 55) [7]. In addition, a propensity score matching (PSM) analysis was performed based on age, prostate volume, Charlson Comorbidity Index score, and preoperative total IPSS, which could indicate significant differences between the two groups. Twelve patients each were evaluated in the PUL and TUEB groups.

G*Power was used to set the following parameters: effect size = 0.6, power = 0.8, and significance level = 0.05 [8]. Approximately 24 participants were required to achieve the required sample size. In contrast, when G*Power was used with the PUL (n = 25) and TUEB (n = 55) groups, the effect size was 0.7, power was 0.8, and significance level was 0.05, and the calculated power was 0.81.

Parametric continuous data are presented as means ± standard deviations and non-parametric continuous data as median with IQR. Student’s t-test (paired for pre vs. post and unpaired for PUL vs. TUEB) or the Mann-Whitney U test (PUL vs. TUEB) was used to compare continuous data between the groups. The categorical variables were used the chi-squared test (Charlson comorbidity index 0 vs. 1 or more). All statistical analyses were performed using IBM SPSS Statistics for Mac version 28.0 (IBM Japan, Tokyo, Japan). *P*-values were reported in accordance with the guidelines outlined by Assel et al. [9], and *p* values < 0.05 were considered statistically significant.

## Results

**Fig. 1:**
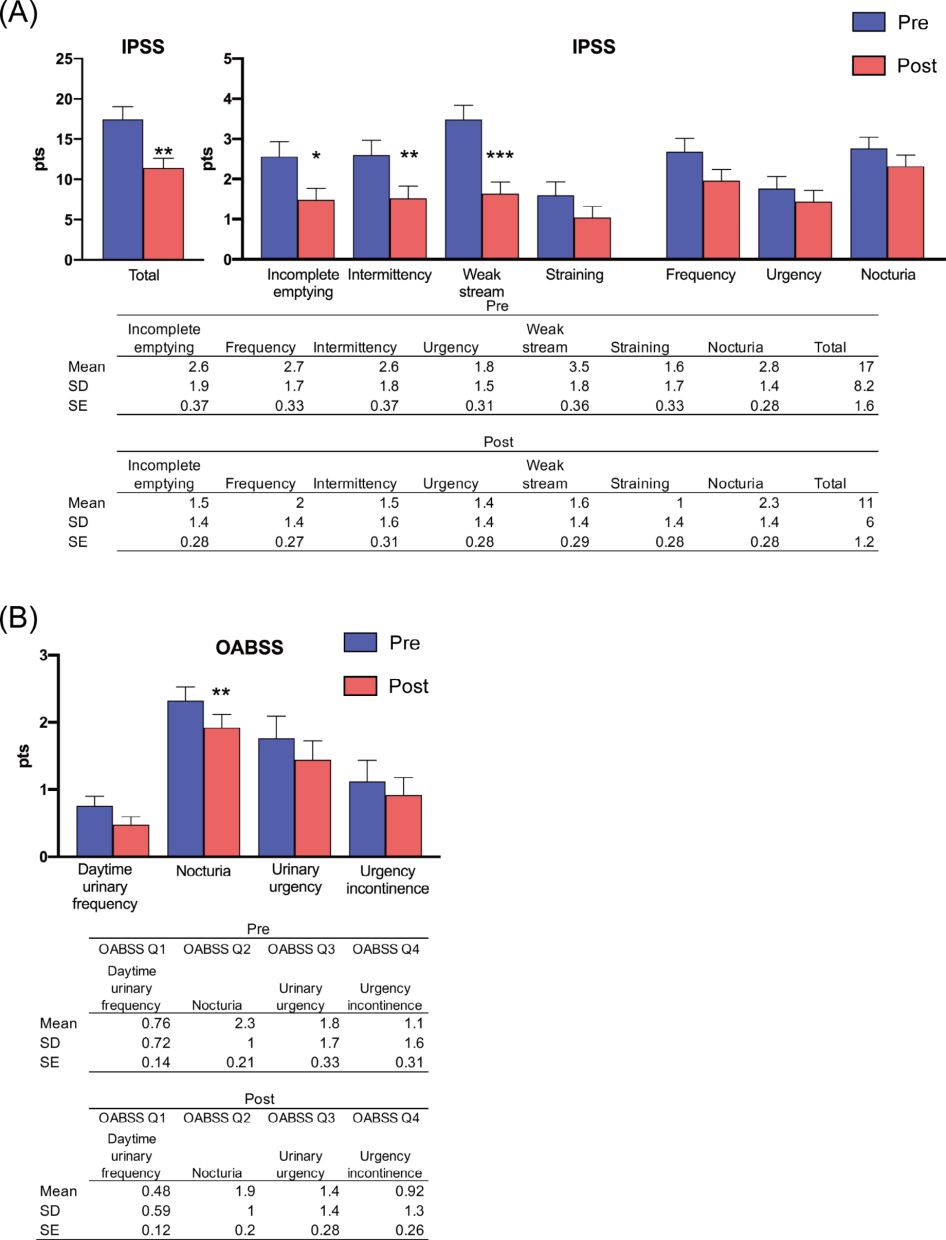
(**A**) Alterations in the total IPSS and domain scores prior to (pre) and 1 month after (post) PUL. (**B**) Comparison of OABSS scores before (pre) and 1 month after (post) the (^*^The results were statistically significant, with *p* < 0.05). ^*^p < 0.05, ^**^p < 0.01 ^***^p ≤ 0.001 compared with preoperative score, as determined using the paired t-test). The table below presents the means, standard deviations, and standard errors for each group. Error bars in the bar graph represent standard errors Abbreviations: IPSS = International Prostate Symptom Score, PUL = prostatic urethral lift, OABSS = Overactive Bladder Symptom Score, SD = standard deviation, SE = standard error

**Fig. 2:**
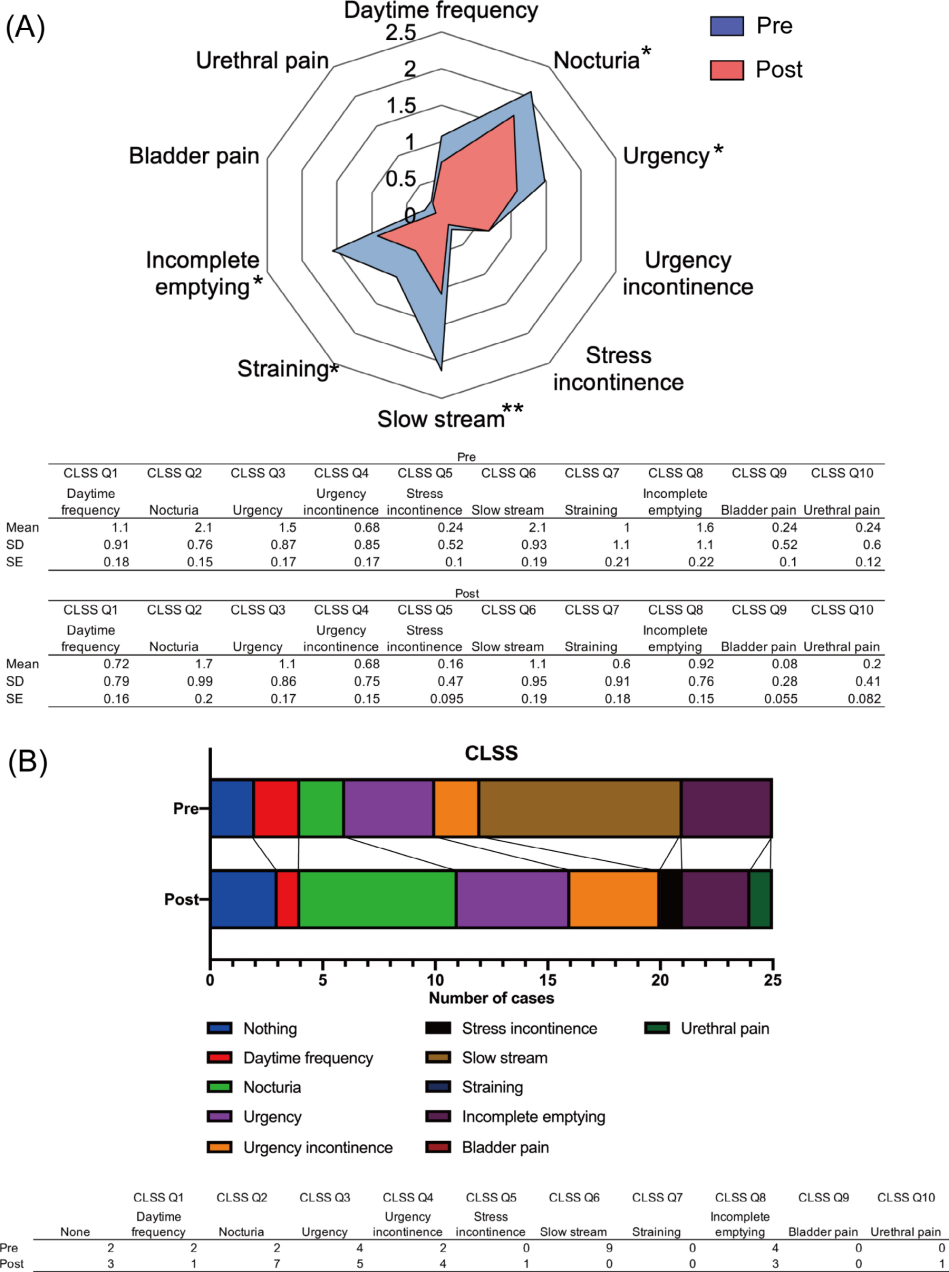
(**A**) Differences in CLSS domain scores before (pre) and 1 month after (post) PUL (^*^p < 0.05, ^**^The preoperative score was significantly different from the postoperative score [*p* < 0.01, paired t-test]). The table below presents the means, standard deviations, and standard errors for each group. Error bars in the bar graph represent standard errors. (**B**) Variations in the number of domains most influential to quality of life selected before and 1 month after PUL. The table below presents the number of patients who selected each symptom for each group Abbreviations: CLSS = Core Lower Urinary Tract Symptom Score, PUL = prostatic urethral lift, SD = standard deviation, SE = standard error

**Fig. 3:**
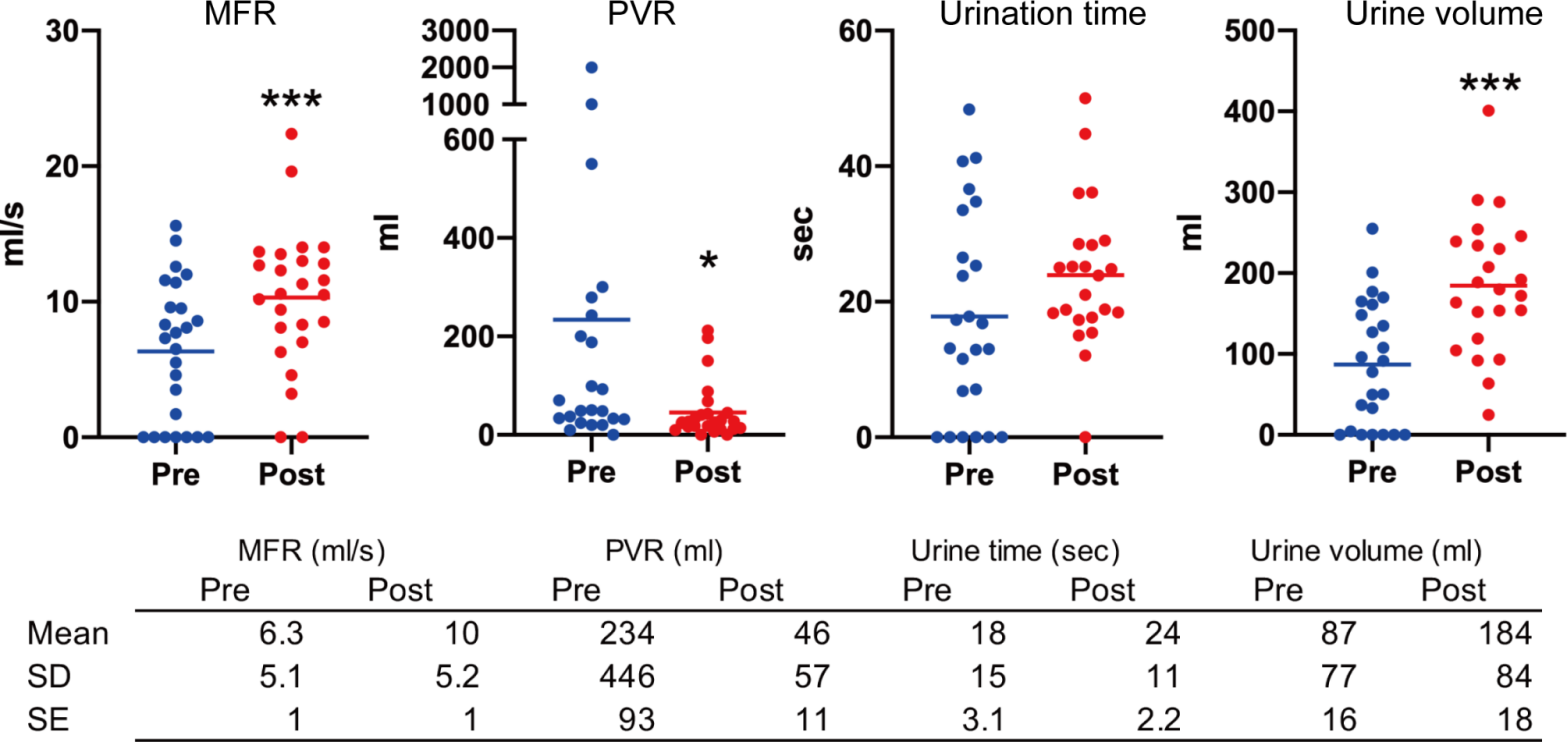
(**A**) Changes in MFR, PVR, urination time, and urine volume 1 month after PUL (^*^p < 0.01, ^***^p < 0.001 vs. preoperative score). The table below presents the means, standard deviations, and standard errors for each group. The horizontal bars indicate the means for each group Abbreviations: MFR = maximum flow rate, PVR = post-void residual, PUL = prostatic urethral lift, SD = standard deviation, SE = standard error

The mean age of the participants was 74.6 years, prostate volume was 47.5 ml, G8 score was 14.1, and there were 13 cases (52%) had a Charlson comorbidity index score of 1 or higher. Antithrombotic drugs were administered in 15 (60%) patients, and 8 (32%) patients had preoperative urinary retention. The median operative time was 15.0 min. The average number of implants used was 4.2 (Table 1). The PUL procedure significantly improved the total IPSS (*p* = 0.002) and, specifically, the urinary domain of the IPSS (incomplete emptying, *p* = 0.041; intermittency, *p* = 0.005; and weak stream, *p* = 0.001) (Fig. 1A; Table 2). Although we saw some evidence of improvement in the straining (*p* = 0.065) and storage domains (frequency, *p* = 0.062; urgency, *p* = 0.11; and nocturia, *p* = 0.17), differences between groups did not meet conventional levels of statistical significance (Fig. 1A; Table 2). The same pattern was observed in the OABSS (Q1: daytime frequency, *p* = 0.050; Q3: urinary urgency, *p* = 0.13; Q4: urgency incontinence, *p* = 0.3), with a significant improvement in Q2: nocturia domain (*p* = 0.022) (Fig. 1B). The CLSS demonstrated significant improvements in the voiding domain (Q6: slow stream, *p* = 0.002; Q7: straining, *p* = 0.024; Q8: incomplete emptying, *p* = 0.046) and in some storage domains such as nocturia (Q2, *p* = 0.038) and urgency (Q3, *p* = 0.038) (Fig. 2A and B; Table 2). Regarding selecting the most influential quality of life domain in Pre-CLSS, although a higher proportion of patients selected “slow stream” (9/25 patients), none of the patients selected this domain after PUL. There was an increase in the selection of “nocturia” (from 2 to 7/25 patients), “urgency” (from 4 to 5/25 patients), “urgency incontinence” (from 2 to 4/25 patients), and “stress incontinence” (from 0 to 1/25 patients) after PUL (Fig. 2A and B; Table 2). In addition, the PUL procedure significantly improved MFR (*p* < 0.001), PVR (*p* = 0.039), and urine volume (*p* < 0.001) (Fig. 3).

**Table 1:** Patient characteristics (n = 25)

Parameters	
Age (years), average (SD^a^)	74.6 (7.0)
Body mass index, average (SD)	23.3 (2.8)
Prostate volume (ml), median (IQR^b^)	40 (35–61)
Geriatric 8 score, median (IQR)	13 (13–16)
Number of Charlson Comorbidity Index score 1 or more (%)	13 (52)
Number of patients receiving antithrombotic drugs (%)	15 (60)
Number of preoperative urinary retention cases (%)	8 (32)
Operative time (minutes), median (IQR)	15.0 (11.5–20.0)
Number of implants, average (SD)	4.2 (1.4)

^a^SD = standard deviation

^b^IQR = interquartile range

**Table 2:** Comparison of preoperative and postoperative differences

Post – pre	Mean difference	SD^f^	*p* value
IPSS^a^ Q1	–1.08	2.50	0.041
IPSS Q2	–0.72	1.84	0.062
IPSS Q3	–1.08	1.75	0.005
IPSS Q4	–0.32	0.99	0.11
IPSS Q5	–1.84	2.49	0.001
IPSS Q6	–0.56	1.45	0.065
IPSS Q7	–0.44	1.58	0.17
Total IPSS	–6.04	8.55	0.002
OABSS^b^ Q1	–0.28	0.68	0.050
OABSS Q2	–0.40	0.82	0.022
OABSS Q3	–0.32	1.03	0.13
OABSS Q4	–0.20	1.08	0.3
CLSS^c^ Q1	–0.36	0.86	0.047
CLSS Q2	–0.40	0.91	0.038
CLSS Q3	–0.40	0.91	0.038
CLSS Q4	0.00	0.71	1
CLSS Q5	–0.08	0.57	0.4
CLSS Q6	–1.04	1.46	0.002
CLSS Q7	–0.44	0.92	0.024
CLSS Q8	–0.64	1.52	0.046
CLSS Q9	–0.16	0.47	0.10
CLSS Q10	–0.04	0.73	0.7
MFR^d^	4.0	3.9	< 0.001
PVR^e^	–185.4	405.3	0.039
Urine volume	95.5	66.8	< 0.001
Urination time	4.8	13.0	0.097

^a^IPSS = International Prostate Symptom Score

^b^OABSS = Overactive Bladder Symptom Score

^c^CLSS = Core Lower Urinary Tract Symptom Score

^d^MFR = maximum flow rate

^e^PVR = post-void residual

^f^SD = standard deviation

**Fig. 4:**
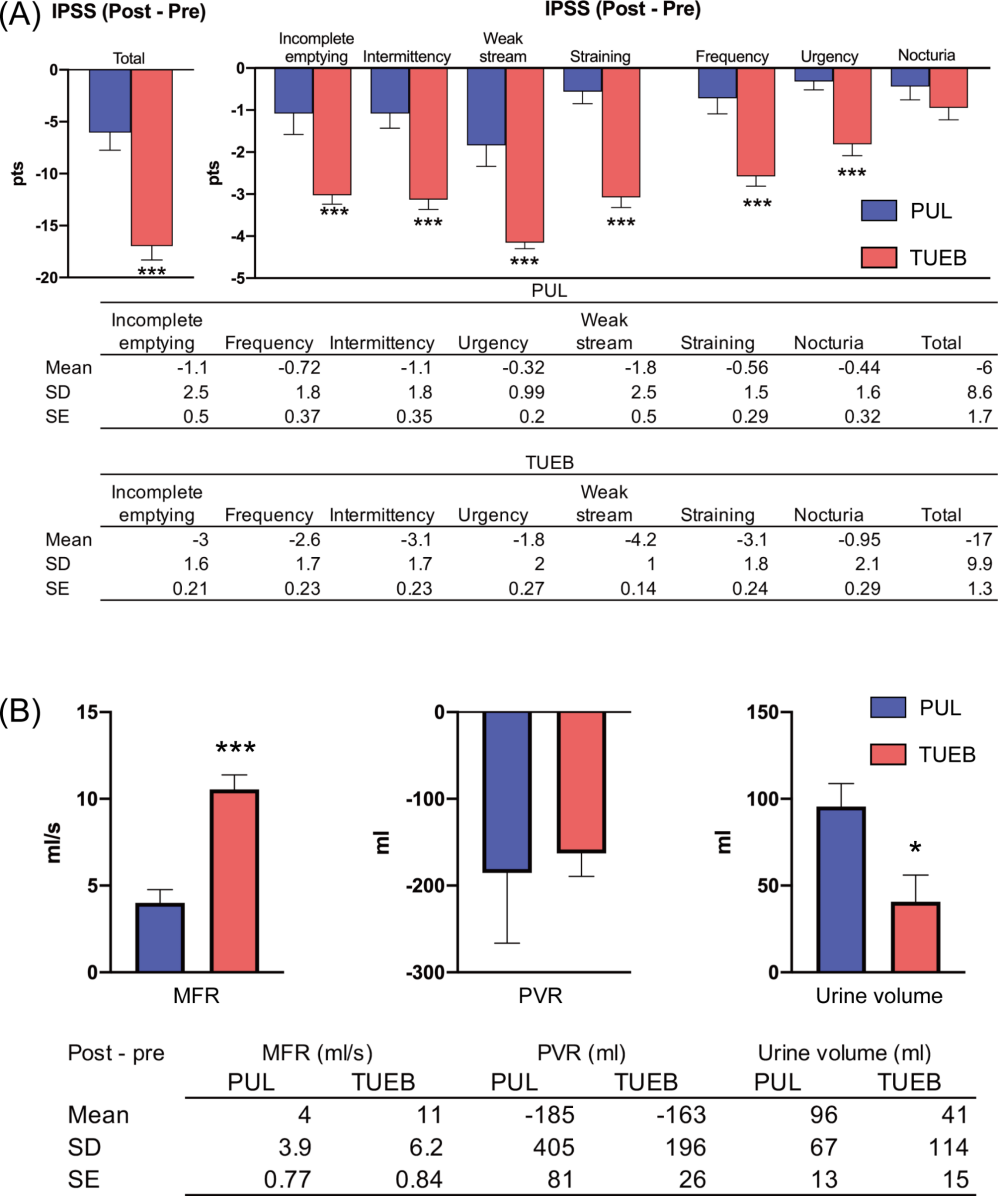
Pre- and postoperative changes in each IPSS domain (**A**) and MFR, PVR, and urine volume (**B**) (^*^p < 0.01, ^***^p < 0.001 vs. preoperative score). The table below presents the means, standard deviations, and standard errors for each group. Error bars in the bar graph represent standard errors Abbreviations: IPSS = International Prostate Symptom Score, MFR = maximum flow rate, PVR, post-void residual, SD = standard deviation, SE = standard error

The PUL group (n = 25) included significantly older adults (*p* = 0.002) and had a higher Charlson Comorbidity Index score (*p* = 0.048) than the TUEB group (n = 55). Prostate volume was significantly greater (*p* < 0.001) and all domains of the total IPSS, except nocturia (*p* = 0.6), were more severe in the TUEB group than in the PUL group (Table 3). There were no statistically significant differences in preoperative MFR and PVR between the two groups (both *p* = 0.7). The operative time was significantly shorter for PUL than for TUEB (*p* < 0.001). Neither procedure resulted in severe perioperative adverse events; however, the incidence of stress urinary incontinence was significantly lower in the PUL group than in the TUEB group (*p* = 0.022; Table 4). In terms of the IPSS, the postoperative total IPSS and voiding symptom domains in the TUEB group were significantly lower than those in the PUL group (Table 3), whereas there were no significant differences in the storage symptom domains between the two groups (Table 3). However, regarding the change in the postoperative IPSS compared to the preoperative IPSS, the total score and the scores of all domains, except nocturia, for TUEB were drastically improved compared to those for PUL (Fig. 4A). In addition, TUEB showed significantly higher postoperative MFR improvement than PUL (*p* < 0.001); however, there were no statistically significant differences in PVR changing between the groups (*p* = 0.7). The PUL group had significantly higher urine volume improvement than the TUEB group (*p* = 0.028, Table 3; Fig. 4B). After propensity score matching based on patient age, prostate volume, Charlson Comorbidity Index scores, and preoperative total IPSS, which resulted in a reduced number of cases in each group, we found no statistically significant difference in postoperative IPSS outcomes, except for the weak stream domain and total score, and the postoperative MFR between PUL and TUEB (Table 5).

**Table 3:** Comparison of each parameter of PULa or TUEBb

Parameters	PUL	TUEB	*p* value	Cohen’s d
Age (years), mean (SD^c^)	74.6 (7.0)	69.2 (7.0)	0.002	0.76
Prostate volume (ml), median (IQR^d^)	40 (35–61)	64.1 (48–87.5)	< 0.001	-
Number of Charlson Comorbidity Index score 1 or more (%)	13 (52)	16 (29)	0.048	-
Preoperative				
MFR^e^ (ml/s), mean (SD)	6.9 (5)	6.6 (3.1)	0.7	0.096
PVR^f^ (ml), mean (SD)	233.9 (446.4)	208.8 (194.8)	0.7	0.081
Urine volume, mean (SD)	86.9 (76.9)	141.1 (91.7)	0.028	–0.62
IPSS^g^ Q1, mean (SD)	2.6 (1.9)	3.8 (1.7)	0.005	–0.71
IPSS Q2, mean (SD)	2.7 (1.7)	4 (1.4)	0.001	–0.83
IPSS Q3, mean (SD)	2.6 (1.8)	3.7 (1.7)	0.01	–0.60
IPSS Q4, mean (SD)	1.8 (1.5)	3.2 (1.9)	0.003	–0.77
IPSS Q5, mean (SD)	3.5 (1.8)	4.5 (1.1)	0.005	–0.71
IPSS Q6, mean (SD)	1.6 (1.7)	3.3 (2)	< 0.001	–0.89
IPSS Q7, mean (SD)	2.8 (1.4)	3 (1.4)	0.6	–0.10
Total IPSS, mean (SD)	17.4 (8.1)	22.7 (9.9)	0.025	-0.56
Operative time (minutes), median (IQR)	15.0 (11.5–20.0)	138.0 (100.2–169.2)	< 0.001	–
Postoperative				
MFR (ml/s), mean (SD)	11.2 (4.4)	17.3 (6.8)	0.01	–0.98
PVR (ml), mean (SD)	45.8 (57.3)	50.8 (56.5)	0.7	–0.08
Urine volume, mean (SD)	184.5 (84.5)	164.4 (84.6)	0.3	0.23
IPSS Q1, mean (SD)	1.5 (1.5)	0.8 (0.9)	0.01	0.71
IPSS Q2, mean (SD)	2 (1.4)	1.4 (1.2)	0.061	0.48
IPSS Q3, mean (SD)	1.6 (1.6)	0.5 (1)	0.01	0.91
IPSS Q4, mean (SD)	1.5 (1.4)	1.2 (1.2)	0.3	0.25
IPSS Q5, mean (SD)	1.7 (1.5)	0.5 (0.7)	0.01	1.18
IPSS Q6, mean (SD)	1.1 (1.4)	0.3 (0.6)	0.01	0.87
IPSS Q7, mean (SD)	2.4 (1.5)	1.9 (1.5)	0.19	0.33
Total IPSS, mean (SD)	11.4 (6.0)	5.2 (4.2)	< 0.001	1.25

^a^PUL = prostatic urethral lift

^b^TUEB = bipolar transurethral enucleation of the prostate

^c^SD = standard deviation

^d^IQR = interquartile range

^e^MFR = maximum flow rate

^f^PVR = post-void residual

^g^IPSS = International Prostate Symptom Score

**Table 4:** Intraoperative and postoperative complications

Parameters	PUL^a^ (n = 25)	TUEB^b^ (n = 55)	*p* value
Transfusions	0 (0%)	0 (0%)	-
Infections	0 (0%)	3 (5.4%)	0.23
Urinary retention	2 (8.0%)	2 (3.6%)	0.4
De novo OAB	0 (0%)	5 (9.0%)	0.11
Transient stress urinary incontinence	1 (4.0%)	14 (25%)	0.022
Urethral stricture	0 (0%)	4 (7.2%)	0.16

^a^PUL = prostatic urethral lift

^b^TUEB = bipolar transurethral enucleation of the prostate

^c^OAB = overactive bladder

**Table 5:** Comparison of each parameter of PULa or TUEBb after propensity score matching based on age, prostate volume, Charlson Comorbidity Index score, and preoperative total IPSS

Parameters	PUL (n = 12)	TUEB (n = 12)	*p* value	Cohen’s d
Age (years), mean (SD^c^)	71.8 (6.4)	71.1 (7.5)	0.8	0.095
Prostate volume (ml), median (IQR^d^)	59.5 (39.5–65)	63.5 (41.7–66.6)	0.5	-
Number of Charlson Comorbidity Index score 1 or more (%)	4 (33)	5 (41)	0.6	-
Preoperative				
MFR^e^ (ml/s), mean (SD)	7.4 (4)	7.3 (2.3)	0.9	-0.31
PVR^f^ (ml), mean (SD)	102.5 (158.8)	253.8 (312.8)	0.15	0.62
Urine volume, mean (SD)	75.6 (50.3)	192.4 (129.5)	0.01	1.26
IPSS^g^ Q1, mean (SD)	3.2 (1.8)	3.6 (1.2)	0.6	0.22
IPSS Q2, mean (SD)	2.6 (1.7)	3.8 (1.6)	0.13	0.66
IPSS Q3, mean (SD)	2.9 (2.0)	2.9 (1.9)	0.9	–0.008
IPSS Q4, mean (SD)	1.6 (1.4)	2.1 (1.9)	0.5	0.25
IPSS Q5, mean (SD)	3.6 (1.6)	4.4 (0.9)	0.23	0.52
IPSS Q6, mean (SD)	1.8 (1.7)	2.3 (1.8)	0.5	0.25
IPSS Q7, mean (SD)	2.5 (1.3)	2.6 (1.2)	0.9	0.13
Total IPSS, mean (SD)	18.5 (8.5)	18 (10.2)	0.8	-0.53
Operative time (minutes), median (IQR)	14 (11.2–21)	138.5 (124.2–153.5)	< 0.001	–
Postoperative				
MFR (ml/s), mean (SD)	12.5 (2.9)	15.1 (5.6)	0.18	–0.59
PVR (ml), mean (SD)	43.2 (51.3)	88 (91.9)	0.16	–0.6
Urine volume, mean (SD)	180.8 (40.5)	154.9 (104.8)	0.4	0.33
IPSS Q1, mean (SD)	1.5 (1.3)	0.8 (0.9)	0.18	0.58
IPSS Q2, mean (SD)	1.8 (1.1)	1.4 (1.0)	0.3	0.38
IPSS Q3, mean (SD)	1.5 (1.4)	0.8 (1.6)	0.2	0.45
IPSS Q4, mean (SD)	1.2 (1.2)	0.6 (0.5)	0.13	0.67
IPSS Q5, mean (SD)	1.7 (1.5)	0.6 (0.6)	0.042	0.92
IPSS Q6, mean (SD)	0.9 (1.4)	0.2 (0.6)	0.16	0.62
IPSS Q7, mean (SD)	2 (1.2)	1.8 (0.7)	0.6	0.18
Total IPSS, mean (SD)	10.7 (6.7)	5.5 (4.1)	0.035	0.91

^a^PUL = prostatic urethral lift

^b^TUEB = bipolar transurethral enucleation of the prostate

^c^SD = standard deviation

^d^IQR = interquartile range

^e^MFR = maximum flow rate

^f^PVR = post-void residual

^g^IPSS = International Prostate Symptom Score

## Discussion

BPH is commonly observed in older adults [10], and prostate volume tends to increase with age [11]. Surgical intervention is necessary for patients with a large prostate volume or BPH-related complications such as bladder or recurrent urinary tract stones. Furthermore, medications for BPH often have a high discontinuation rate over time, with approximately half of the patients discontinuing treatment within 2 years [2]. Moreover, α1-blocker monotherapy or discontinuation of oral therapy is a risk factor for surgery [2]. In Japan, which currently has an aging population, there is an anticipated increase in the number of patients requiring surgical intervention for BPH.

However, older adults often present with various complications, including geriatric syndromes. Geriatric syndromes encompass several conditions, such as osteoporosis and dementia, which have a multi-organ effect. As a result, surgeries in older patients are often considered high risk owing to the presence of comorbidities and aging. Screening tools such as the Charlson Comorbidity Index and G8 have been developed to assess and identify high-risk cases. The Charlson Comorbidity Index assigns scores based on the presence of comorbidities, and higher total scores are generally associated with poorer prognoses [12]. G8 is a functional assessment tool used to evaluate activities of daily living and prognosis in the older population. A G8 score < 14 is generally indicative of a poor prognosis [13]. In this study, compared with the TUEB group, the PUL group had a lower mean G8 score of 14, indicating higher risk and poorer prognosis among older patients. Additionally, the PUL group included older patients with a higher Charlson Comorbidity Index score compared with the TUEB group.

In 2011, PUL was the first surgical procedure for which outcomes were reported on a global scale [5]. In the initial report, a group of 19 Australian patients showed favorable IPSS outcomes 1 year after undergoing PUL. Subsequently, the efficacy and safety of PUL were evaluated in a multicenter, prospective, randomized, controlled blinded trial [14]. A 5-year follow-up report in 2017 showed an improvement in IPSS within 2 weeks postoperatively, which was maintained for 5 years [15]. Consistent with previous reports, the current study observed improvements in IPSS but also used two symptom questionnaires developed in Japan, the OABSS [16] for OAB symptoms and the CLSS [17] for core or key symptoms in several conditions, for a more detailed study. The CLSS questionnaire focuses on 10 key symptoms selected from a list of 25 established by the International Continence Society Standardization Committee. In the present study, a notable finding was the improvement in storage domains, including nocturia, in the PUL group; however, it was also noted that more patients had selected nocturia as the most influential quality of life domain. This improvement was not captured by the IPSS questionnaire alone but was evident when using the CLSS and OABSS questionnaires. The IPSS, OABSS, and CLSS have distinct focuses, timeframes, and evaluation objectives. Specifically, although the IPSS assesses symptoms over the past month, both the OABSS and CLSS evaluate symptoms over the past week. This difference allows the OABSS and CLSS to more precisely reflect the patient’s condition 1 month postoperatively, whereas the IPSS might be influenced by the immediate effects of the surgery. The IPSS primarily evaluates symptom frequency, excluding nocturia, whereas the OABSS evaluates symptoms based on specific counts over 1 week, again excluding nocturia and daytime frequency. Additionally, the OABSS assesses incontinence, which is not part of the IPSS, thus allowing for the diagnosis and grading of overactive bladder [18]. However, the CLSS differs from both the OABSS and IPSS because it does not evaluate the patient’s condition based on specific counts or frequencies; instead, it gauges the level of discomfort that the patient feels by using answers such as “none,” “occasionally,” “sometimes,” and “always” [17, 19]. Furthermore, although the IPSS includes four questions related to voiding, the CLSS, which aims to provide a comprehensive assessment, includes only three questions. The CLSS omits questions about interrupted streams and includes two questions about pain. We evaluated the responses to the IPSS Q7, OABSS Q2, and CLSS Q2 by seven patients who reported nocturia as their most bothersome postoperative symptom, as determined by the CLSS. Notably, all seven patients experienced nocturia before surgery; however, their postoperative outcomes were disparate. Three patients exhibited improvement, one patient experienced worsened symptoms, and the remaining three patients experienced no change (Supplementary Table). These findings suggest that as other symptoms improve, nocturia may have more of an impact on the patient’s quality of life and could be considered the most influential quality of life domain.

Therefore, the incorporation of the CLSS and OABSS allows for a more comprehensive and straightforward assessment of these symptoms.

TURP has traditionally been considered a reference surgical method for treating BPH [20]. Prospective randomized controlled trials comparing PUL and TURP have been conducted [21]. Both groups demonstrated significant improvements in symptoms and MFR. Although TURP was superior to PUL, particularly in terms of improvement in IPSS and MFR, postoperative worsening of urinary incontinence was observed in the TURP group but not in the PUL group [22]. TUEB shows better efficacy than TURP for treating BPH with a large prostate volume. TUEB is superior to TURP in the complete endoscopic resection of prostatic adenomas but with a longer operative time and significantly higher postoperative incidence of urethral stricture and stress urinary incontinence [7]. Compared with outcomes previously reported at our institution for TUEB [7], TUEB was better than PUL in terms of IPSS and Qmax in younger and less complicated patients. However, urine volume was increased in PUL, and the postoperative PVR was similar in both the groups. No significant complications were observed in any patient. Furthermore, when patient backgrounds are standardized for key preoperative characteristics in PUL and TUEB, the therapeutic outcomes of both procedures are considered closely aligned. This finding suggests that PUL is minimally invasive and offers several benefits.

This study had some limitations. The limitations of our study include the lack of strict control of variables typical in prospective studies, which may result in information bias due to subtle differences in patient characteristics. Furthermore, our patient cohort, derived from a single institution, may not accurately represent a broader population, potentially affecting the external validity of our findings. Although the number of patients was small, Cohen’s d values were sufficiently large to provide sufficient power, and the questionnaire used and comparison with the TUEB have not been reported previously, making this the first study in Japan. In Japan, PUL is indicated only for elderly patients who are at high risk, and conducting a sexual function assessment of such patients can be challenging. Therefore, this study did not include sexual function evaluations. Another limitation of this study was the short follow-up period of only 1 month. This procedure was introduced in Japan in April 2023, and it is currently limited to high-risk cases, resulting in a low number of cases nationwide since its implementation. Therefore, longer follow-up periods were not possible. As a result, Japan-specific outcome data was not available. Although future studies involving longer durations and larger cohorts are needed, this study may provide evidence to support the adoption of this new treatment modality in Japan.

In conclusion, this study reports the short-term results of PUL and safely demonstrates its efficacy in high-risk older patients. It is anticipated that the introduction of minimally invasive surgery in Japan will lead to an increase in the number of BPH surgeries, which are currently declining.

### Electronic supplementary material

Below is the link to the electronic supplementary material.

Supplementary Material 1

## Data Availability

The data that support the findings of this study are available from the corresponding author, upon reasonable request.
