# Supplementary material for: Initial experience with prostatic urethral lift versus enucleation of the prostate: a retrospective comparative study
Source: BMC Urol. 2023 Nov 18;23:188. doi: 10.1186/s12894-023-01366-8 (PMC10657556; doi:10.1186/s12894-023-01366-8)
Supplement: Supplementary file 1 — Supplementary Material 1 [file 12894_2023_1366_MOESM1_ESM.docx]

**Supplementary Table**

Evaluation of seven patients who selected nocturia as the “most influential quality of life domain symptom” postoperatively according to the CLSS

| Patient ID | CLSS Q2  pre | IPSS Q7  pre | OABSS Q2  pre | CLSS Q2  post-pre | IPSS Q7  post-pre | OABSS Q2  post-pre |
| --- | --- | --- | --- | --- | --- | --- |
| 1 | 3 | 5 | 3 | -3 | -2 | -2 |
| 7 | 3 | 5 | 3 | -1 | -3 | -1 |
| 10 | 2 | 3 | 3 | 1 | 0 | 0 |
| 12 | 3 | 5 | 3 | 0 | 0 | 0 |
| 17 | 2 | 2 | 0 | 0 | 0 | 2 |
| 18 | 2 | 2 | 2 | 0 | 0 | 0 |
| 26 | 3 | 3 | 3 | -1 | -1 | -1 |

^a^CLSS = Core Lower Urinary Tract Symptom Score

^b^Pre = preoperatively

^c^Post = postoperatively
